# Hypnotism v. Dypsomania

**Published:** 1889-11-09

**Authors:** 


### Hypnotism v. Dipsomania.

The treatment of dipsomania has not hitherto been attended with great success. The habitual drunkard cannot be shut up in a retreat against his will, and his will is usually to go free. Then if he consent to a period of seclusion, the terms asked at most of our homes for inebriates are so high as to be prohibitive to all but a small class. He is not fit to be placed in an ordinary asylum, for he is mentally the superior of the lunatics with whom he associates, while morally he is sunk to a far lower level than they. A recent writer in the *Manchester Weekly Times* quotes the superintendent of a large asylum as saying, "You have no right to impose the company of such liars and mischief-makers upon respectable lunatics"; and this certainly represents the feeling of a large class, both of asylum officials and inmates.

What, then, is to be done with the habitual drunkard? To leave him free to ruin his family and achieve a gradual suicide seems less than the duty of a Christian nation, and the short terms of imprisonment which a magistrate may inflict by way of punishment have rarely a deterrent effect. While we are reflecting on the fact that the present Habitual Drunkards' Act expires in 1890, and wondering what better means of restraint and cure can be devised in a succeeding enactment, there comes the news that a new treatment has been tried, with all appearance of success, by the Rev. Arthur Tooth. Mr. Tooth achieved notoriety some years ago by church services "higher" than his parishioners at Hatcham liked, and since then he had almost disappeared from view, till he turned up as a minister to the diseased minds and bodies of dipsomaniacs. His method is that of the hypnotism which from fickle France and mystic, unchanging India simultaneously attacks the stolid British sceptic. The drunkard is thrown into a hypnotic sleep, and then Mr. Tooth talks to him, argues with him on the folly and wickedness of drunkenness, and extracts from him expressions of abhorrence of alcohol. This abhorrence is supposed to remain with the hypnotic subject after his return to consciousness, and affect his future actions. Mr. Tooth gives demonstrations of his method in presence of spectators, and makes no pretence to mystery in the treatment. It seems evident that, so far as immediate results go, the hypnotic treatment of dipsomania is sufficiently satisfactory. But immediate results are hardly things to judge by. In fact, it is one of the characteristics of drunkards to speak of alcohol with the utmost horror when they are themselves comparatively sober. They can point out its ravages in others, can dilate on the folly of yielding to the craving for it, will even refuse to partake of it in presence of others, and then sneak off to gratify their craving in private. Many really feel a horror of it when they are recovering from a severe bout of drinking. They are guilty of no equivocation when they declare that they hate the sight and smell of it; for the time they really revolt from it. You often meet with such cases. They keep straight for weeks and months; then, when their friends are hoping for a permanent reformation, they break down. But while it lasts, their repentance is sincere, their horror of drink and drunkenness unfeigned. Is it possible to bring about such feelings, and the resolves that follow them, by outside means, and to render them permanent? This is what hypnotism tries to do.

It is a question of moral influence—the ascendancy of a strong mind over a weak one. That it is real while it lasts it would be futile to deny; but the question which cannot be settled by one visit to Croydon is how long this influence does last. It would be necessary to follow up a case for many months after the treatment had ceased, to be sure that the resolutions formed under the controlling influence remain when that is gone. It is hardly possible that this should be so in all cases; but in some, where mind and body are not hopelessly diseased with the craving for drink, the change of ideas, the development of distaste in place of liking, may give a new turn to the mind, and bring about a distinct reformation.

